# Supplementary material for: Fostering active choice to empower behavioral change to reduce cardiovascular risk: A web-based randomized controlled trial
Source: PLoS One. 2024 Aug 1;19(8):e0304897. doi: 10.1371/journal.pone.0304897 (PMC11293644; doi:10.1371/journal.pone.0304897)
Supplement: S1 File — (PDF) [file pone.0304897.s001.pdf]

Additional File 1. SPSS RESEACH CODE MAIN ANALYSES

```
*****************************************************************
************************* ACTIVE CHOICE *************************
*****************************************************************

EXAMINE VARIABLES=MEAN_ActiveChoice
 /PLOT BOXPLOT HISTOGRAM
 /COMPARE GROUPS
 /STATISTICS DESCRIPTIVES
 /CINTERVAL 95
 /MISSING PAIRWISE
 /NOTOTAL.

EXAMINE VARIABLES=MEAN_ActiveChoice BY GROUP
 /PLOT BOXPLOT HISTOGRAM
 /COMPARE GROUPS
 /STATISTICS DESCRIPTIVES
 /CINTERVAL 95
 /MISSING PAIRWISE
 /NOTOTAL.

REGRESSION
 /DESCRIPTIVES MEAN STDDEV CORR SIG N
 /MISSING PAIRWISE
 /STATISTICS COEFF OUTS CI(95) R ANOVA CHANGE
 /CRITERIA=PIN(.05) POUT(.10)
```

```
 /NOORIGIN
 /DEPENDENT MEAN_ActiveChoice
 /METHOD=ENTER GROUP
 /RESIDUALS HISTOGRAM(ZRESID) NORMPROB(ZRESID).

**********************************************************************
************************* INTENTION STRENGTH *************************
**********************************************************************

EXAMINE VARIABLES=IntentionStrength_Highest
 /PLOT BOXPLOT HISTOGRAM
 /COMPARE GROUPS
 /STATISTICS DESCRIPTIVES
 /CINTERVAL 95
 /MISSING PAIRWISE
 /NOTOTAL.

EXAMINE VARIABLES=IntentionStrength_Highest BY GROUP
 /PLOT BOXPLOT HISTOGRAM
 /COMPARE GROUPS
 /STATISTICS DESCRIPTIVES
 /CINTERVAL 95
 /MISSING PAIRWISE
 /NOTOTAL.

REGRESSION
 /DESCRIPTIVES MEAN STDDEV CORR SIG N
```

```
  /MISSING PAIRWISE
  /STATISTICS COEFF OUTS CI(95) R ANOVA CHANGE
  /CRITERIA=PIN(.05) POUT(.10)
  /NOORIGIN
  /DEPENDENT IntentionStrength_Highest
  /METHOD=ENTER GROUP
  /RESIDUALS HISTOGRAM(ZRESID) NORMPROB(ZRESID).

*********************************************************************
************************** COMMITMENT ******************************
*********************************************************************

EXAMINE VARIABLES=MEAN_Commitment
  /PLOT BOXPLOT HISTOGRAM
  /COMPARE GROUPS
  /STATISTICS DESCRIPTIVES
  /CINTERVAL 95
  /MISSING PAIRWISE
  /NOTOTAL.

PPLOT
  /VARIABLES=MEAN_Commitment
  /NOLOG
  /NOSTANDARDIZE
  /TYPE=Q-Q
  /FRACTION=BLOM
  /TIES=MEAN
```

```
  /DIST=NORMAL.

EXAMINE VARIABLES=MEAN_Commitment BY GROUP
  /PLOT BOXPLOT HISTOGRAM
  /COMPARE GROUPS
  /STATISTICS DESCRIPTIVES
  /CINTERVAL 95
  /MISSING PAIRWISE
  /NOTOTAL.

REGRESSION
  /DESCRIPTIVES MEAN STDDEV CORR SIG N
  /MISSING PAIRWISE
  /STATISTICS COEFF OUTS CI(95) R ANOVA CHANGE
  /CRITERIA=PIN(.05) POUT(.10)
  /NOORIGIN
  /DEPENDENT MEAN_Commitment
  /METHOD=ENTER GROUP
  /RESIDUALS HISTOGRAM(ZRESID) NORMPROB(ZRESID).

****************************************************************************
************************** INTENTION (4 CATEGORIES) **************************
****************************************************************************

CROSSTABS
  /TABLES=GROUP BY Intention_4CAT
  /FORMAT=AVALUE TABLES
```

```
  /STATISTICS=CHISQ
  /CELLS=COUNT ROW
  /COUNT ROUND CELL.

RECODE GROUP (0=1) (1=0) INTO Group_andersom.
VARIABLE LABELS  Group_andersom 'Group coded the other way around'.
EXECUTE.

NOMREG Intention_4CAT (BASE=LAST ORDER=ASCENDING) BY Group_andersom
  /CRITERIA CIN(95) DELTA(0) MXITER(100) MXSTEP(5) CHKSEP(20) LCONVERGE(0)
PCONVERGE(0.000001)
    SINGULAR(0.00000001)
  /MODEL
  /STEPWISE=PIN(.05) POUT(0.1) MINEFFECT(0) RULE(SINGLE) ENTRYMETHOD(LR)
REMOVALMETHOD(LR)
  /INTERCEPT=INCLUDE
  /PRINT=PARAMETER SUMMARY LRT CPS STEP MFI.

RECODE Intention_4CAT (4=0) (1=1) (2=1) (3=1) (SYSMIS=SYSMIS) INTO
Intention_NoChange_vs_Change.
VARIABLE LABELS  Intention_NoChange_vs_Change 'Dichotomized variable for intention'.
EXECUTE.

CROSSTABS
  /TABLES=Group_andersom BY Intention_NoChange_vs_Change
  /FORMAT=AVALUE TABLES
  /STATISTICS=RISK
  /CELLS=COUNT
  /COUNT ROUND CELL
```

```
  /METHOD=EXACT TIMER(5).

****************************************************************************
************************** LIFESTYLE INTENTION: DIET  **************************
****************************************************************************

CROSSTABS
  /TABLES=GROUP BY Lifestyle_Intention_Diet
  /FORMAT=AVALUE TABLES
  /STATISTICS=CHISQ
  /CELLS=COUNT ROW
  /COUNT ROUND CELL.

LOGISTIC REGRESSION VARIABLES Lifestyle_Intention_Diet
  /METHOD=ENTER GROUP
  /CONTRAST (GROUP)=Indicator(1)
  /PRINT=CI(95)
  /CRITERIA=PIN(0.05) POUT(0.10) ITERATE(20) CUT(0.5).

**********************************************************************
**************** LIFESTYLE INTENTION: PHYSICAL ACTIVITY *******************
**********************************************************************

CROSSTABS
  /TABLES=GROUP BY Lifestyle_Intention_PA
  /FORMAT=AVALUE TABLES
  /STATISTICS=CHISQ
```

```
  /CELLS=COUNT ROW
  /COUNT ROUND CELL.

LOGISTIC REGRESSION VARIABLES Lifestyle_Intention_PA
  /METHOD=ENTER GROUP
  /CONTRAST (GROUP)=Indicator(1)
  /PRINT=CI(95)
  /CRITERIA=PIN(0.05) POUT(0.10) ITERATE(20) CUT(0.5).

*****************************************************************
******************* LIFESTYLE INTENTION: OTHER ***********************
*****************************************************************

CROSSTABS
  /TABLES=GROUP BY Lifestyle_Intention_Other
  /FORMAT=AVALUE TABLES
  /STATISTICS=CHISQ
  /CELLS=COUNT ROW
  /COUNT ROUND CELL.

LOGISTIC REGRESSION VARIABLES Lifestyle_Intention_Other
  /METHOD=ENTER GROUP
  /CONTRAST (GROUP)=Indicator(1)
  /PRINT=CI(95)
  /CRITERIA=PIN(0.05) POUT(0.10) ITERATE(20) CUT(0.5).
```

```
*************************************************************************

************************** SELF-EFFICACY *********************************

*************************************************************************


EXAMINE VARIABLES= SelfEfficacy_Highest
  /PLOT BOXPLOT HISTOGRAM
  /COMPARE GROUPS
  /STATISTICS DESCRIPTIVES
  /CINTERVAL 95
  /MISSING PAIRWISE
  /NOTOTAL.

EXAMINE VARIABLES= SelfEfficacy_Highest BY GROUP
  /PLOT BOXPLOT HISTOGRAM
  /COMPARE GROUPS
  /STATISTICS DESCRIPTIVES
  /CINTERVAL 95
  /MISSING PAIRWISE
  /NOTOTAL.

REGRESSION
  /DESCRIPTIVES MEAN STDDEV CORR SIG N
  /MISSING PAIRWISE
  /STATISTICS COEFF OUTS CI(95) R ANOVA CHANGE
  /CRITERIA=PIN(.05) POUT(.10)
  /NOORIGIN
```

```
  /DEPENDENT  SelfEfficacy_Highest
  /METHOD=ENTER GROUP
  /RESIDUALS HISTOGRAM(ZRESID) NORMPROB(ZRESID).

**********************************************************************
************************* RESPONSE EFFICACY ******************************
**********************************************************************

* RESPONSE EFFICACY CONCERNING LIFESTYLE CHANGE:

EXAMINE VARIABLES= ResponseEfficacy_1
  /PLOT BOXPLOT HISTOGRAM
  /COMPARE GROUPS
  /STATISTICS DESCRIPTIVES
  /CINTERVAL 95
  /MISSING PAIRWISE
  /NOTOTAL.

EXAMINE VARIABLES= ResponseEfficacy_1 BY GROUP
  /PLOT BOXPLOT HISTOGRAM
  /COMPARE GROUPS
  /STATISTICS DESCRIPTIVES
  /CINTERVAL 95
  /MISSING PAIRWISE
  /NOTOTAL.
REGRESSION
  /DESCRIPTIVES MEAN STDDEV CORR SIG N
```

```
  /MISSING PAIRWISE
  /STATISTICS COEFF OUTS CI(95) R ANOVA CHANGE
  /CRITERIA=PIN(.05) POUT(.10)
  /NOORIGIN
  /DEPENDENT  ResponseEfficacy_1
  /METHOD=ENTER GROUP
  /RESIDUALS HISTOGRAM(ZRESID) NORMPROB(ZRESID).

* RESPONSE EFFICACY CONCERNING MEDICATION USE:

EXAMINE VARIABLES= ResponseEfficacy_2
  /PLOT BOXPLOT HISTOGRAM
  /COMPARE GROUPS
  /STATISTICS DESCRIPTIVES
  /CINTERVAL 95
  /MISSING PAIRWISE
  /NOTOTAL.

EXAMINE VARIABLES= ResponseEfficacy_2 BY GROUP
  /PLOT BOXPLOT HISTOGRAM
  /COMPARE GROUPS
  /STATISTICS DESCRIPTIVES
  /CINTERVAL 95
  /MISSING PAIRWISE
  /NOTOTAL.

REGRESSION
```

```
  /DESCRIPTIVES MEAN STDDEV CORR SIG N
  /MISSING PAIRWISE
  /STATISTICS COEFF OUTS CI(95) R ANOVA CHANGE
  /CRITERIA=PIN(.05) POUT(.10)
  /NOORIGIN
  /DEPENDENT  ResponseEfficacy_2
  /METHOD=ENTER GROUP
  /RESIDUALS HISTOGRAM(ZRESID) NORMPROB(ZRESID).

************************************************************************
************************** AUTONOMOUS MOTIVATION *************************
************************************************************************

EXAMINE VARIABLES= MEAN_AutonomousMotivation
  /PLOT BOXPLOT HISTOGRAM
  /COMPARE GROUPS
  /STATISTICS DESCRIPTIVES
  /CINTERVAL 95
  /MISSING PAIRWISE
  /NOTOTAL.

* The distribution is skewed due to a ceiling effect.

PPLOT
  /VARIABLES=MEAN_AutonomousMotivation
  /NOLOG
  /NOSTANDARDIZE
```

```
 /TYPE=Q-Q
 /FRACTION=BLOM
 /TIES=MEAN
 /DIST=NORMAL.
```

* We checked the Q-Q plot with the statistician. We performed a log transformation:

```
PPLOT
 /VARIABLES=MEAN_AutonomousMotivation
 /LN
 /NOSTANDARDIZE
 /TYPE=Q-Q
 /FRACTION=BLOM
 /TIES=MEAN
 /DIST=NORMAL.

EXAMINE VARIABLES= MEAN_AutonomousMotivation BY GROUP
 /PLOT BOXPLOT HISTOGRAM
 /COMPARE GROUPS
 /STATISTICS DESCRIPTIVES
 /CINTERVAL 95
 /MISSING PAIRWISE
 /NOTOTAL.

COMPUTE LN_MEAN_AutonomousMotivation = LN(MEAN_AutonomousMotivation).

REGRESSION
```

```
 /MISSING LISTWISE
 /STATISTICS COEFF OUTS CI(95) R ANOVA
 /CRITERIA=PIN(.05) POUT(.10)
 /NOORIGIN
 /DEPENDENT LN_MEAN_AutonomousMotivation
 /METHOD=ENTER GROUP
 /SCATTERPLOT=(*SDRESID ,LN_MEAN_AutonomousMotivation)
 /RESIDUALS HISTOGRAM(ZRESID) NORMPROB(ZRESID).

********************************************************************
************************* KNOWLEDGE *********************************
********************************************************************

EXAMINE VARIABLES= Knowledge_SUM_4items
 /PLOT BOXPLOT HISTOGRAM
 /COMPARE GROUPS
 /STATISTICS DESCRIPTIVES
 /CINTERVAL 95
 /MISSING PAIRWISE
 /NOTOTAL.

EXAMINE VARIABLES= Knowledge_SUM_4items BY GROUP
 /PLOT BOXPLOT HISTOGRAM
 /COMPARE GROUPS
 /STATISTICS DESCRIPTIVES
 /CINTERVAL 95
 /MISSING PAIRWISE
```

```
  /NOTOTAL.


PLUM Knowledge_SUM_4items BY GROUP
  /CRITERIA=CIN(95) DELTA(0) LCONVERGE(0) MXITER(100) MXSTEP(5) PCONVERGE(1.0E-6)
SINGULAR(1.0E-8)
  /LINK=LOGIT
  /PRINT=FIT PARAMETER SUMMARY.


PLUM Knowledge_SUM_4items BY Group_andersom
  /CRITERIA=CIN(95) DELTA(0) LCONVERGE(0) MXITER(100) MXSTEP(5) PCONVERGE(1.0E-6)
SINGULAR(1.0E-8)
  /LINK=LOGIT
  /PRINT=FIT PARAMETER SUMMARY.


********************************************************************************
************************** COGNITIVE RISK PERCEPTION ****************************
********************************************************************************


EXAMINE VARIABLES= RiskPerception_Cognitive
  /PLOT BOXPLOT HISTOGRAM
  /COMPARE GROUPS
  /STATISTICS DESCRIPTIVES
  /CINTERVAL 95
  /MISSING PAIRWISE
  /NOTOTAL.


EXAMINE VARIABLES= RiskPerception_Cognitive BY GROUP
  /PLOT BOXPLOT HISTOGRAM
```

```
 /COMPARE GROUPS
 /STATISTICS DESCRIPTIVES
 /CINTERVAL 95
 /MISSING PAIRWISE
 /NOTOTAL.

REGRESSION
 /DESCRIPTIVES MEAN STDDEV CORR SIG N
 /MISSING PAIRWISE
 /STATISTICS COEFF OUTS CI(95) R ANOVA CHANGE
 /CRITERIA=PIN(.05) POUT(.10)
 /NOORIGIN
 /DEPENDENT RiskPerception_Cognitive
 /METHOD=ENTER GROUP
 /RESIDUALS HISTOGRAM(ZRESID) NORMPROB(ZRESID).

*******************************************************************************
************************** AFFECTIVE RISK PERCEPTION *****************************
*******************************************************************************

EXAMINE VARIABLES= MEAN_AffectiveRiskPerception
 /PLOT BOXPLOT HISTOGRAM
 /COMPARE GROUPS
 /STATISTICS DESCRIPTIVES
 /CINTERVAL 95
 /MISSING PAIRWISE
 /NOTOTAL.
```

```
EXAMINE VARIABLES= MEAN_AffectiveRiskPerception BY GROUP
  /PLOT BOXPLOT HISTOGRAM
  /COMPARE GROUPS
  /STATISTICS DESCRIPTIVES
  /CINTERVAL 95
  /MISSING PAIRWISE
  /NOTOTAL.

REGRESSION
  /DESCRIPTIVES MEAN STDDEV CORR SIG N
  /MISSING PAIRWISE
  /STATISTICS COEFF OUTS CI(95) R ANOVA CHANGE
  /CRITERIA=PIN(.05) POUT(.10)
  /NOORIGIN
  /DEPENDENT MEAN_AffectiveRiskPerception
  /METHOD=ENTER GROUP
  /RESIDUALS HISTOGRAM(ZRESID) NORMPROB(ZRESID).
```
