# Supplementary material for: Fostering active choice to empower behavioral change to reduce cardiovascular risk: A web-based randomized controlled trial
Source: PLoS One. 2024 Aug 1;19(8):e0304897. doi: 10.1371/journal.pone.0304897 (PMC11293644; doi:10.1371/journal.pone.0304897)
Supplement: S2 File — (PDF) [file pone.0304897.s002.pdf]

The Active Choice (AC) intervention

Note: The following pages show the webpages that were shown to participants. The original intervention was in Dutch; the current document has been translated into English for publication.

In the real web-based intervention, participants' heart age was tailored to their actual age. In the current document, we have used an age of 65 years and a corresponding heart age of 78 years as an example.
In addition, the last exercise of the intervention was tailored to participants' responses in the real web-based intervention. In the current document, we have shown the icons that correspond to scores of 6 and above.

Imagine this…

Imagine you visit you general practitioner.
Your general practitioner explains:

> "Your blood pressure is high. Based on your blood pressure, cholesterol, age, and gender, I calculated the age of your heart*.
>
> You are now **65 years** old; however, your heart and blood vessels are comparable to someone **13 years** older.
> So your heart age is:
>
> **78 years**
>
> This means you are at increased risk of dying from cardiovascular disease."

Note that this heart age was devised for this study and therefore does not really belong to you.

*This was calculated using the Heart Age calculation tool of the Dutch Heart Foundation. A healthy diet, sufficient physical activity and low stress levels are also important for a healthy heart, but not accurate enough to calculate heart age. Therefore, these factors were not included in the calculation.

Your heart age is

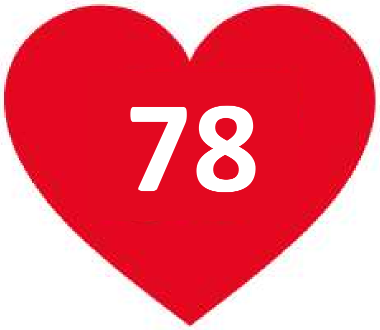

Causes and consequences

What do I notice?

- High blood pressure damages the blood vessels, but you usually don't notice it.

Causes

- If cardiovascular disease runs in your family, you are more likely to get it as well. You cannot influence that yourself.
- The risk of cardiovascular disease also increases as you get older.
- Smoking, an unhealthy diet and little physical activity are also a major cause of cardiovascular disease. You can influence that yourself.

Consequences

- The blood vessels are being damaged. This can lead to cardiovascular disease, such as a stroke or heart attack. Possible consequences are paralysis or death.

Your heart age is

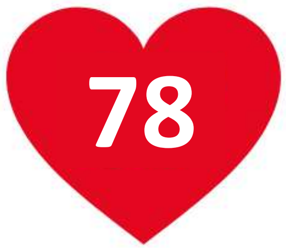

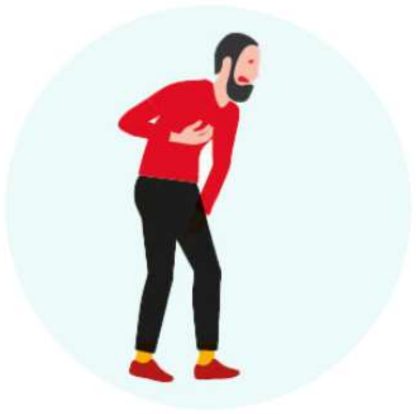

How do I want to cope with this?

It is up to you how you want to cope with an increased risk of cardiovascular disease.

You can reduce the risk by changing your lifestyle or by taking medication. You can also choose to do both. Another option is to not change; this will not reduce the risk of cardiovascular disease.

More information about each option will be presented on the next pages. Please read this information carefully.

Option 1: Lifestyle change

Option 2: Medication use

Option 3: Lifestyle change and medication use

Option 4: No change

Option 1: Lifestyle change

What does this mean?

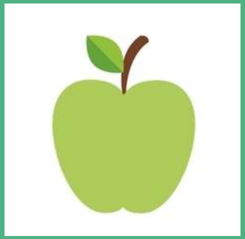

Healthy diet
A healthy diet consists of plenty of vegetables, fruits, legumes (i.e., beans, peas), whole grains, nuts and fish. Red/processed meat, pretzels, biscuits and sweet products are not recommended.

Sufficient physical activity
Sufficient physical activity means engaging in at least 2,5 hours of physical activity per week, spread over multiple days. For example: walking, cycling, running, playing football, fitness, dancing, or swimming. Sitting for long periods of time is not recommended.

Do not smoke
Not smoking means not smoking yourself and avoiding second-hand smoke.

Advantages

- It improves your physical health (think of more energy and fitness, for example).
- It improves your mental health (think of better coping with stress, for example).
- It reduces your risk of cardiovascular disease and diabetes. Here's what it does to your heart age of 78:

HEALTHY DIET: 74 = 4 years younger*

PHYSICAL ACTIVITY: 76 = 2 years younger*

*Note: The changes in heart age are a general estimate; they cannot be determined very precisely. You cannot simply add up the changes over the years, because they are interrelated. If healthy eating leads to 4 years younger and sufficient physical activity to 2 years younger, then the combination does not automatically lead to 6 years younger. However, the more you do to a healthy lifestyle, the smaller your risk of cardiovascular disease will generally be.

Disadvantages

- Changing your lifestyle requires motivation and discipline.
- A healthier diet and more physical activity can take more time and cost more money.

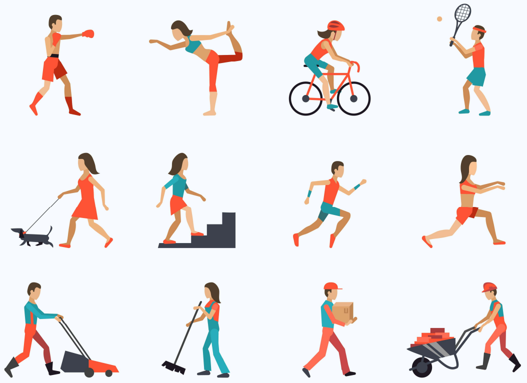

Which of these advantages and/or disadvantages do you consider most important? Select them by clicking the star. You may select multiple stars.

Option 2: Medication use

What does this mean?

- Every day you take one or multiple pills to reduce your blood pressure.
- You often take these pills for the rest of your life.
- You may discuss with you general practitioner which medication is best for you.

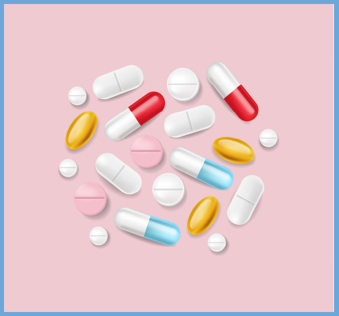

Advantages

☆ Blood pressure lowering medications reduce your risk of cardiovascular disease. Here's what it does to your heart age of 78:

MEDICATION USE: 75 = 3 years younger*

*Note: The changes in heart age are a general estimate; they cannot be determined very precisely.

Disadvantages

☆ Blood pressure lowering medications may cause side effects. Examples include:

- Dizziness
- Blurred vision
- Coughing
- Stomach and intestinal complaints

For a particular medication that is commonly used to treat high blood pressure, these first three side effects occur very frequently and the last one frequently[i].

☆ These medications cost money. They are reimbursed by the insurance company, but you still pay an amount.

☆ You must remember to take your medicines every day.

? **Which of these advantages and/or disadvantages do you consider most important? Select them by clicking the star. You may select multiple stars.**

[i] *(Info button): 'Frequently' means that more than 10 out of 100 people will suffer from this. 'Very frequently' means that 1 to 10 out of 100 individuals will suffer from this.*

Option 3: Lifestyle change and medication use

What does this mean?

A healthy lifestyle means:

- A healthy diet
- Sufficient physical activity
- No smoking

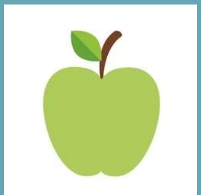

Medication use means:

- Every day you take one or multiple pills to reduce your blood pressure.
- You often take these pills for the rest of your life.

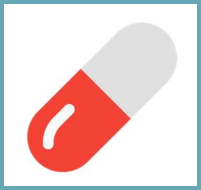

Advantages

Your risk of cardiovascular disease reduces even faster if you both change your lifestyle and take medication. Here's what it does to your heart age of 78:

HEALTHY DIET:	74	= 4 years younger*
PHYSICAL ACTIVITY	76	= 2 years younger*
MEDICATION USE:	75	= 3 years younger*

*Note: The changes in heart age are a general estimate; they cannot be determined very precisely. You cannot simply add up the changes over the years, because they are interrelated. If healthy eating leads to 4 years younger and sufficient physical activity to 2 years younger, then the combination does not automatically lead to 6 years younger. However, the more you do to a healthy lifestyle, the smaller your risk of cardiovascular disease will generally be.

Disadvantages

Changing your lifestyle and taking medication requires even more motivation and discipline.

A healthier diet and more physical activity can take more time and cost more money.

Blood pressure lowering medications may cause side effects.

These medications cost money. They are reimbursed by the insurance company, but you still pay an amount.

Which of these advantages and/or disadvantages do you consider most important? Select them by clicking the star. You may select multiple stars.

Option 4: No change

What does this mean?

This means that you do not change your lifestyle nor take medication.

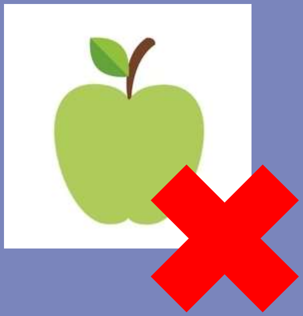

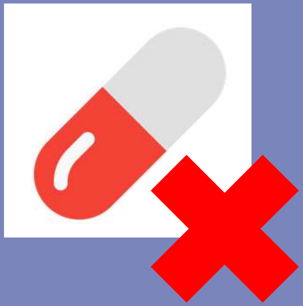

Advantages

- It will not take you additional time or effort, and there are no additional costs.
- You will not experience side effects.

Disadvantages

- Your general physical and mental health will not improve.
- Your risk of getting cardiovascular diseases remains high. This puts you at a higher risk of premature death.

Which of these advantages and/or disadvantages do you consider most important? Select them by clicking the star. You may select multiple stars.

Exercise: What I think is important

If you had to choose what to do, it would be good to think more about what is important to you.

To what extent do you agree with the following statements?
Please indicate this on a scale of 1 (totally disagree) to 10 (totally agree).

		Totally disagree									Totally agree
1.	It is important to me to live as long as possible in good health	1	2	3	4	5	6	7	8	9	10
2.	It is important to me to spend time and energy on improving my health	1	2	3	4	5	6	7	8	9	10
3.	It would bother me if I would have to change my diet and physical activity behavior	1	2	3	4	5	6	7	8	9	10
4.	It would bother me to depend on medication	1	2	3	4	5	6	7	8	9	10
5.	It would bother me to get the substances of medication in my body	1	2	3	4	5	6	7	8	9	10
6.	I would accept the risk of side effects of medication	1	2	3	4	5	6	7	8	9	10

Exercise: What I think is important

Your scores from the previous exercise are presented below. Moreover, icons are now added to show what choice option fits each statement.

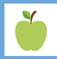 = Lifestyle change

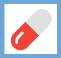 = Medication use

Example: Lifestyle and medication use fit your answer if you scored a 6 or higher on the first statement.
Note: Not changing your lifestyle and not taking medication is also always a choice option.

		Totally disagree									Totally agree	
1.	It is important to me to live as long as possible in good health	1	2	3	4	5	6	7	8	9	10	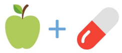
2.	It is important to me to spend time and energy on improving my health	1	2	3	4	5	6	7	8	9	10	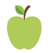
3.	It would bother me if I would have to change my diet and physical activity behavior	1	2	3	4	5	6	7	8	9	10	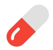
4.	It would bother me to depend on medication	1	2	3	4	5	6	7	8	9	10	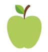
5.	It would bother me to get the substances of medication in my body	1	2	3	4	5	6	7	8	9	10	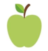
6.	I would accept the risk of side effects from medication	1	2	3	4	5	6	7	8	9	10	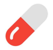

Please check how many apple and pill icons are shown next to the statements. Which choice option seems to best fit your answers? Is it ‘lifestyle change’, ‘medication use’, both, or none of them? Please think about this.
