# Supplementary material for: Fostering active choice to empower behavioral change to reduce cardiovascular risk: A web-based randomized controlled trial
Source: PLoS One. 2024 Aug 1;19(8):e0304897. doi: 10.1371/journal.pone.0304897 (PMC11293644; doi:10.1371/journal.pone.0304897)
Supplement: S3 File — (PDF) [file pone.0304897.s003.pdf]

The Passive Choice (PC) control condition

Note: The following pages show the webpages that were shown to participants. The original intervention was in Dutch; the current document has been translated into English for publication.

Imagine this…

Imagine you visit you general practitioner.
Your general practitioner explains:

> "Your blood pressure is high. Based on your blood pressure, cholesterol, age, and gender, I calculated your risk of dying from cardiovascular disease within 10 years*. This risk is:
>
> **8%**
>
> This means you are at increased risk of dying from cardiovascular disease."

Note that this result was devised for this study and therefore does not really belong to you.

*A healthy diet, sufficient physical activity and low stress levels are also important for good health, but not accurate enough to calculate the risk of cardiovascular disease. Therefore, these factors were not included in the calculation.

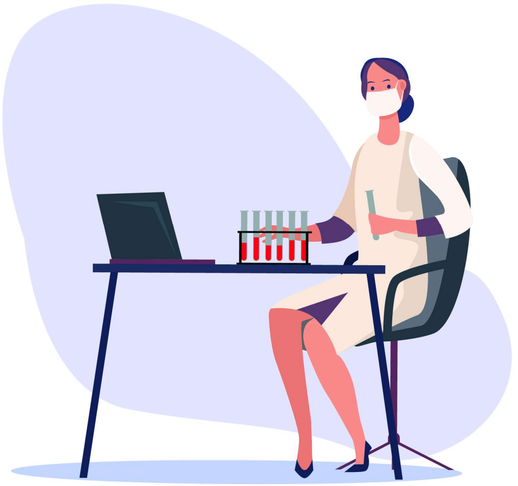

How can I lower the risk?

Your general practitioner advises you to follow a healthy lifestyle to lower you risk: “That means: A healthy diet, sufficient physical activity and no smoking”.

In addition, your general practitioner suggests that you take medication to lower your blood pressure.

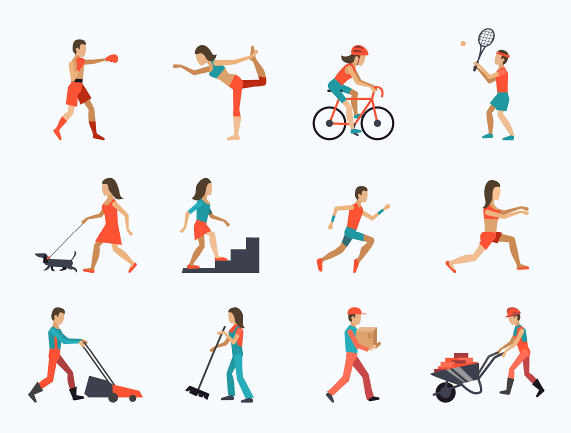

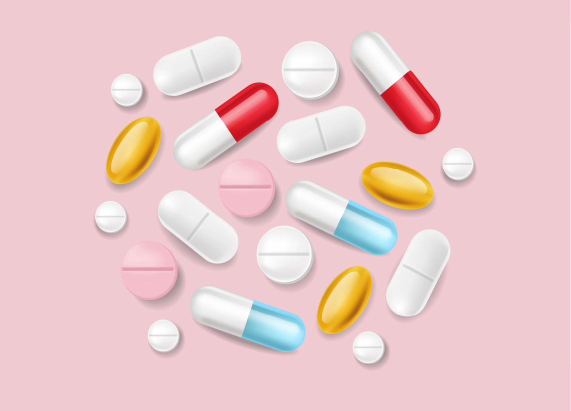

What does this mean?

What does a healthy lifestyle mean?

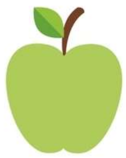

- **Healthy diet**

A healthy diet consists of plenty of vegetables, fruits, legumes (i.e., beans, peas), whole grains, nuts and fish. Red/processed meat, pretzels, biscuits and sweet products are not recommended.

- **Sufficient physical activity**

Sufficient physical activity means engaging in at least 2,5 hours of physical activity per week, spread over multiple days. For example: walking, cycling, running, playing football, fitness, dancing, or swimming. Sitting for long periods of time is not recommended.

- **Do not smoke**

Not smoking means not smoking yourself and avoiding second-hand smoke.

What does medication use mean?

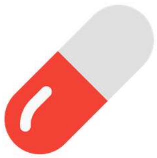

- Every day you take one or multiple pills to reduce your blood pressure.
- You often take these pills for the rest of your life.
- You may discuss with you general practitioner which medication is best for you.
